# Trading people versus trading time: What is the difference?

**DOI:** 10.1186/1478-7954-3-10

**Published:** 2005-11-10

**Authors:** Laura J Damschroder, Todd R Roberts, Christine C Goldstein, Molly E Miklosovic, Peter A Ubel

**Affiliations:** 1 VA Health Service Research & Development Center of Excellence, VA Ann Arbor Healthcare System, Ann Arbor, MI. USA; 2 Division of General Internal Medicine, University of Michigan Medical School, Ann Arbor, Michigan, USA; 3 The Center for Behavioral and Decision Sciences in Medicine, University of Michigan and VA Ann Arbor Healthcare System, Ann Arbor, MI USA; 4 Department of Psychology, University of Michigan, Ann Arbor, MI USA

## Abstract

**Background:**

Person trade-off (PTO) elicitations yield different values than standard utility measures, such as time trade-off (TTO) elicitations. Some people believe this difference arises because the PTO captures the importance of distributive principles other than maximizing treatment benefits. We conducted a qualitative study to determine whether people mention considerations related to distributive principles other than QALY-maximization more often in PTO elicitations than in TTO elicitations and whether this could account for the empirical differences.

**Methods:**

64 members of the general public were randomized to one of three different face-to-face interviews, thinking aloud as they responded to TTO and PTO elicitations. Participants responded to a TTO followed by a PTO elicitation within contexts that compared either: 1) two life-saving treatments; 2) two cure treatments; or 3) a life-saving treatment versus a cure treatment.

**Results:**

When people were asked to choose between life-saving treatments, non-maximizing principles were more common with the PTO than the TTO task. Only 5% of participants considered non-maximizing principles as they responded to the TTO elicitation compared to 68% of participants who did so when responding to the PTO elicitation. Non-maximizing principles that emerged included importance of equality of life and a desire to avoid discrimination. However, these principles were less common in the other two contexts. Regardless of context, though, participants were significantly more likely to respond from a societal perspective with the PTO compared to the TTO elicitation.

**Conclusion:**

When lives are at stake, within the context of a PTO elicitation, people are more likely to consider non-maximizing principles, including the importance of equal access to a life-saving treatment, avoiding prejudice or discrimination, and in rare cases giving treatment priority based purely on the position of being worse-off.

## Background

Preliminary results from this study were presented in a poster session at the 2002 AcademyHealth Conference on June 23, 2002, and final results were presented at the 25th Annual Meeting of the Society Medical Decision Making on October 2003.

Cost-effectiveness analyses (CEAs) show how to maximize the number of quality adjusted life-years (QALYs) that can be obtained within a given budget – an approach we refer to as "QALY-maximization." The United States Public Health Service Panel on Cost Effectiveness recommended three methods for measuring preferences in CEAs: the rating scale, the standard gamble, and the time trade-off elicitation technique [1]. Some people argue that none of the three recommended preference measurement techniques are particularly suited for capturing public preferences in allocation or rationing contexts because they support a distributive principle based on maximizing treatment benefits, measured as QALYs, without regard for who receives the benefits. People have described other principles they believe are normatively important to ensure a just distribution of healthcare treatments, including the importance of giving: 1) more weight to patients with a more severe health condition [2-4]; 2) equal weight on saving the lives of people with or without disabilities [4,5]; and 3) sufficiently high priority on treating patients in clear need of beneficial treatments [6-10].

Nord has argued that, "the obvious way to make sure that the QALY procedure captures social preferences for person trade-offs is, of course, to use person trade-off exercises as the basis for scoring health states in the first place" [[3], page 201]. page 201 The person trade-off (PTO) preference elicitation method was proposed to do just that [3,11-16]. In traditional preference measurement methods, such as the time trade-off (TTO) and standard gamble (SG), people are asked, typically from a personal perspective, to state their preference for a health state by imagining they are in that health state but could be returned to perfect health if they lived fewer years (TTO) or won a gamble (SG). In PTO elicitations, people are asked to make tradeoffs between treating different groups of patients who differ by a constellation of attributes. For example, respondents might be asked how many patients would need to be cured of moderate leg pain to be equally good as curing 100 patients of severe shortness of breath.

The debate about the relative merits of traditional utility measures versus the PTO measure is not merely a theoretical one. Numerous studies have shown that preferences elicited by traditional utility measurement methods can differ significantly from those elicited by the PTO method [3,14-21]. Differences are especially striking when lives are at stake. For example, all else being equal, saving the life of someone with a medical disability (e.g. paraplegia) produces fewer QALYs than saving the life of someone who can be returned to perfect health. However, public preferences, as measured by PTO elicitations, place nearly equal value on saving the lives of healthy people and people with paraplegia [22-24].

Why do PTO elicitations yield preference values that are different than values obtained using traditional utility measures? Traditional measures elicit subjective expected utilities for a health condition which are used to set priorities based on principles of QALY-maximization (see for example, Nord [16], Ubel [12], and Olsen [15]). Many people believe that PTO elicitations, in addition to capturing the underlying subjective expected utility for the condition being evaluated, incorporate other distributive principles. Indeed, Salomon and Murray demonstrated how these "distributional concerns" could be derived as weights separate from the "core strength of preference" that is common across many elicitation methods [25]. We conducted this study to gain insight into the factors people consider when responding to PTO elicitations compared to one traditional preference measurement method, the TTO method. We used a think-aloud protocol in face-to-face interviews in which we asked participants to state their reasoning out loud as they responded to both types of elicitations. Specifically, our goal was to determine whether people mention considerations related to distributive principles other than QALY-maximization more often in PTO elicitations than in TTO elicitations and whether this could account for the empirical differences.

## Methods

We recruited a convenience sample of participants from the general public in and around a small Midwestern city in the U.S. Venues included a nearby major metropolitan airport, a local Laundromat, a Veterans Affairs Medical Center, and we recruited some over the phone who traveled on-site for the interview. Willing respondents participated in a semi-structured interview that consisted of one TTO elicitation, followed by multiple PTO elicitations. We obtained participants' strength of preferences for paraplegia, severe shortness of breath, and moderate leg pain using both methods.

### Elicitations

We started the interviews with a TTO elicitation, asking participants to consider two friends, Mr. (Mrs.) Adams and Mr. (Mrs.) Brown who were both 30 years old and who would live with their health condition (e.g. perfect health or paraplegia) for 50 more years, and then die in their sleep. We matched the gender of the imaginary friends to the gender of the participant. Participants responded to one of two TTO elicitations – either making tradeoffs between paraplegia and perfect health or between severe shortness of breath and moderate leg pain. We started off by asking which friend was better off and then, assuming the respondent chose the friend with the less severe condition, asked them to choose between one friend living in the worse-off condition for 50 more years and the other friend living for only a "few more days" with the better-off condition. We chose a lengthy timeframe (50 years) because we believed more participants would willingly trade years over a longer time horizon compared to a shorter time horizon [26,27]. Most TTO elicitations use a personal perspective in which respondents are asked to imagine themselves in the worse-off condition with a chance for a cure if they are willing to live in perfect health for a shorter period of time [28]. However, we used an impersonal perspective (depicting the two friends) because one study found that people were more willing to trade years to improve quality of life with this perspective while only marginally affecting overall values [29] and another study did not find any differences in values when they compared personal versus non-personal perspectives [21].

After the TTO elicitation, participants responded to PTO elicitations in which we asked them to imagine being "on a panel of experts trying to decide between two different medical treatments." We described two alternative treatment programs (see the Appendix) and asked the participants, "Which would you choose or are the choices equally good?" Participants responded to at least two PTO scenarios (as shown in Table 1) in which they were asked to choose between: 1) curing a life-threatening infection in previously health people versus curing a life-threatening infection in patients with paraplegia; 2) curing a life-threatening infection in previously healthy people versus curing patients with a spinal cord injury to prevent paraplegia; or 3) curing patients of severe shortness of breath versus curing patients of moderate leg pain. Our intent was to compare responses from the TTO and PTO elicitations.

**Table 1:** Interview Structure for the Three Experimental Groups

Elicitation	Save-Save Group	Cure-Save Life Group	Cure-Cure Group
TTO	Paraplegia v. Perfect Health	Paraplegia v. Perfect Health	Leg Pain v. Severe Shortness of Breath

PTO	*2 Distractor PTOs*	*1 Distractor PTO*	*1 Distractor PTO*
	
	Cure life-threatening infection in previously healthy patients v. patients with paraplegia	Cure life-threatening infection v. Cure SCI^1^	Leg Pain v. Severe Shortness of Breath

Comparison & Reflection Questions	You have answered two different types of questions in this interview. Health policy experts use both types of questions to research people's opinions about treating illness and disability. Now I want to show you how your answers might be interpreted. I especially want to know what you think about these interpretations		
	In the first part of our interview, we compared Mr./Mrs. Adams and Mr./Mrs. Brown in a type of question called the Time Trade-Off. You said that if Mr./Mrs. Brown lived <*indifference point*> years in perfect health, that he/she would be no better or worse off than Mr./Mrs. Adams, who lived 50 years with <*condition*> A policy expert would interpret that you think one year of perfect health is about the same as <*computed ratio*> years with paraplegia. In other words, you think paraplegia is <*computed ratio*> times worse than perfect health. What do you think about this interpretation?		
	Later in the interview, you made a choice between curing some people of <*condition1*> or curing other people of <*condition2*> in a type of question called the Person Trade-Off. Based on your answer to the Time Trade-Off question, a policy expert might assume you would say that <*predicted PTO*> people would have to be cured of <*condition2*> to make that choice as good as curing 100 people of <*condition1*>. Your actual answer was <*PTO indifference point*>.		
	Which number do you think is a better reflection of your thoughts: the number you actually gave for the person tradeoff or the one that was predicted based on your time tradeoff answer?		
	Do the time tradeoff and person tradeoff questions make you think about different issues?		

^1^SCI = Spinal Cord Injury

We assigned participants to one of three versions of a structured interview. Table 1 highlights the TTO and PTO comparisons that were the focus of each of the three experimental groups. In the *Save-Save *group, our focus of comparison was: 1) a TTO elicitation where participants were asked to trade off years of perfect health versus living 50 years with paraplegia; and 2) a PTO elicitation that asked participants to choose between saving the lives of previously healthy patients or patients with paraplegia. In the *Cure-Save *group, we compared: 1) the same TTO elicitation as the Save-Save group; and 2) a PTO elicitation that asked participants to choose between saving the lives of previously healthy patients and curing spinal cord injury to prevent paraplegia. In the *Cure-Cure *group, we compared: 1) a TTO elicitation where participants were asked to trade off years with moderate leg pain versus living 50 years with severe shortness of breath; and 2) a PTO elicitation that asked participants to choose between curing patients with severe shortness of breath and curing patients with moderate leg pain. We did not want responses to the PTO elicitation to be influenced by responses to the TTO elicitation which was presented first. To help mitigate this possibility, we first asked the participant to respond to PTO "distractor" tasks that presented two unrelated health conditions. We started interviews with the Save-Save version of the interview first. We presented 2 distractor PTO elicitations to participants in this group. Participants in the Save-Save group did not refer back to the earlier TTO elicitation when responding to any of the PTO tasks. We made a decision to reduce to a single distractor task when interviewing the other two groups. Again, in the other two groups, no one made a reference to their earlier TTO response in any of the PTO tasks so we felt that the single distractor task was sufficient.

### Indifference point calculations

In the TTO elicitation, we used a "ping-pong" method [30] to converge on an indifference point; the point at which it was difficult to choose which friend was better off. For example, a participant may have said that Mrs. Adams, who was living with paraplegia for 50 years, was just as well off as Mrs. Brown, who was living in perfect health for 40 years. The utility for e.g. paraplegia can be computed as:



Where U(h_2_) = utility for the health state being evaluated (e.g. paraplegia); t_h1 _= time spent in the less severe health state (e.g. perfect health). In the example, we compute U(h_2_) as 0.8 for paraplegia. A utility cannot be computed for severe shortness of breath because participants traded-off relative to moderate leg pain instead of perfect health or death. In this context, the value is better regarded as a "relative utility." Based on the TTO utility, we predicted a PTO indifference point so we could compare this value to their actual PTO indifference point:



In the example above, the predicted PTO indifference point would be 125. This means that based on the participant's TTO valuation, we expect the participant to be indifferent between saving the lives of 125 people with paraplegia and saving the lives of 100 non-disabled people. We modified the predicted PTO indifference point calculation for participants in the Cure-Save group because the PTO elicitation compares *curing *a spinal cord injury (to prevent paraplegia) versus saving lives. Therefore, the denominator in Equation (2a) is substituted with the utility for *curing *paraplegia rather than the utility for the condition itself, as follows:



Continuing with the example above, the utility for curing paraplegia would be 0.2 and the predicted PTO indifference point for curing spinal cord injury (to prevent paraplegia) would be 500. That is, we would expect the participant to be indifferent between curing 500 patients of spinal cord injury versus saving the lives of 100 non-disabled people.

In the PTO elicitations, we asked participants how many patients would need to be cured in the comparison group (e.g. patients with moderate leg pain) to be equally good as curing 100 patients in the baseline group (e.g. patients with shortness of breath). The indifference point is the number of patients that the participant said was needed in the comparison group for both choices to be equally good. The higher the indifference point, the lower the value placed on curing the comparison group of patients relative to curing the baseline group of patients. We used an open-ended approach to obtain a PTO indifference point rather than a search procedure because the response range was open-ended. People could give as high a number as they wanted. One option, in this situation, might have been to use a titration approach but this approach yields responses that are different depending on the direction values are presented [31,32] and are different than those obtained using a ping-pong procedure [30]. Some participants were unable to state a number without prompting, however. In this situation, we did use a ping-pong approach using 6 billion (roughly equal to the population of the world) as the starting point for the high end and then used a procedure comparable to that used in the TTO elicitation to narrow down to an indifference point.

### Qualitative data collection

Three trained interviewers used a verbal report method to explore the thought process of participants as they responded to the TTO and PTO elicitations. We used a *concurrent think aloud *protocol accompanied by *verbal probing*,[33] and asked participants to literally think aloud while responding to the elicitations, verbalizing any and all thoughts [33-36]. We directed participants to "just say out loud whatever is going through your mind as you answer my questions, even if it seems obvious. There is no right or wrong answer; we just want to hear how you think about these issues." After the interviewer posed each question, she reminded participants to think aloud as they answered the question. We complemented this approach with verbal probing, in which the interviewer prompted participants to expand upon their "think aloud" statements and/or to provide retrospective reports of their thoughts to elicit more complete verbalization [37]. We accomplished this by saying, for example, "Can you tell me a little more about what you were thinking as you came up with that answer?" We used these two methods to produce a combination of concurrent and retrospective reports which, when used together, provided a comprehensive description of participants' thought processes [34].

After the participant responded to the TTO and PTO elicitations, the interviewer used the TTO response to predict what the PTO indifference point would be for the corresponding PTO elicitation. The interviewer presented this prediction and the participant's actual indifference point and asked the participant to reflect on the two values by asking the questions listed at the bottom of Table 1. We audio-recorded and created verbatim transcripts of every interview.

### Quantitative analysis

We compared demographic characteristics across the experimental groups using analysis of variance for continuous variables (i.e. age), and χ^2 ^tests for categorical variables (Fisher's Exact Test for 2 × 2 comparisons) using SPSS Release 10. We coded African American, Hispanic, Native American, and Alaska Native participants as racial minorities. We compared indifference points predicted from TTO responses to actual PTO indifference points using the Wilcoxon signed rank test for paired observations. We also conducted a concordance analysis of qualitative codes for the TTO and PTO elicitations using McNemar's paired comparison tests.

### Qualitative analysis

We conducted a descriptive analysis [38] of the verbatim transcribed interviews. The authors read one-third of the interviews (randomly chosen from each of the three experimental groups) and independently listed themes that arose from the readings. They consolidated themes in a step-wise process (reading and coding in two batches of transcripts) to create a final coding scheme. Once themes and definitions were established, three judges (LJD, TRR, CCG) continued reading through all of the transcripts using the coding scheme to identify themes. We used a consensus approach to resolve differences in coding before proceeding to the next batch of transcripts.

## Results

A total of 75 participants participated in the study. We excluded a total of 11 (15%) participants: 2 interviews were not recorded properly and could not be transcribed; 5 interviews were not completed because participants had to catch their plane or go to their clinic appointment; 2 participants were confused about the questions and their answers were uninterpretable; and 2 participants protested against the questions. One of the "protesters" said the "ultimate answer is that there is no answer" in response to the TTO questions and that the PTO questions were "disgusting...it's like, which child do you love more, with big ears or the one with the small ears." The other protester said that the comparisons presented in both the TTO and PTO elicitations could never be equal. Of the 64 participants included in the analysis, 50% were female and participants had an average of 15 years of education. Table 2 shows participant demographics. Participants in the Save-Save group were marginally younger than participants in the Cure-Save and Cure-Cure groups. The Cure-Save group had a 9% racial minority of participants, while the Save-Save and Cure-Cure groups had 27% and 20%, respectively. We were unable to test for statistical differences in minority representation because of low minority counts in the Cure-Save group.

**Table 2:** Participant Demographic Characteristics

	Save-Save Group (n = 22)	Cure-Save Group (n = 22)	Cure-Cure Group (n = 20)	Overall	p
Minority Status					N/A
Yes	27%	9%	20%	19%	
					
Age – Range	19 – 53	19 – 85	20 – 80	19 – 85	
Mean (SD)	33 (12.9)	45 (16.3)	42 (19.2)	41 (17.2)	0.100
					
Gender					
%Female	46%	55%	50%	50%	0.834
					
Education – Range	9 – 21	11 – 19	12 – 21	9 – 21	
Avg Years (SD)	16 (3.3)	15 (2.2)	16 (2.6)	15 (2.6)	0.411
					
Location					N/A
Metro Airport	50%	0%	0%	17%	
Laundromat	41%	46%	35%	41%	
VAMC	9%	23%	30%	20%	
On-site	0%	32%	35%	22%	

Seven themes emerged through our analysis of the transcribed interviews. Table 4 lists the themes along with illustrative quotes. We organized six of the themes into whether they were consistent with the QALY-maximization objective or not. The seventh theme differentiated participants who responded from a personalized perspective, either in terms of their own experience or mentioning other people they knew well who had experienced the condition. These participants used this prior experience to project what it would be like to live with the condition under consideration themselves. The next three sections present findings for each of the three groups.

**Table 4:** Qualitative Themes and Illustrative Quotes

**Themes consistent with QALY-maximization considerations:**		
Quality of life	References to the condition's impact on quality of life. Could be a positive or negative comment on a societal or individual level.	TTO – "...it sounds to me like Mrs. Adams has ... the more severe symptoms, and I would ... think that...it might impact her quality of life more."
		PTO – "I would choose to cure the people who are severely short of breath because ... I think it so affects the quality of life, every move they make. ...People don't have to walk up hill, they can avoid doing that, they can drive, they can find some other means. ...I don't see that as being something that's completely disabling, whereas I see the severe shortness of breath as completely disabling."
Years of life	Expresses the importance of choosing the option that maximizes the length of life.	TTO – "Probably the one that lives longer is in better shape..."
Non-health benefits	Consideration of the choice that would result in the most benefit in terms of non-health dimensions such as contribution to society, economic contribution, employment, etc.	TTO – "Even though he might have less years to live, he's really capable of doing a lot more."
		PTO – "I guess if you wanted to look at it really, really coldly you could say this group is consuming fewer resources ..."
		
**Themes consistent with equity considerations:**		
Fair Chance	Consideration of giving someone who is worse-off a fair chance even though the benefit may be less than the alternative.	... if ... the non-paraplegic versus the paraplegic, have same length of life span ... probably the quality of life would be a little better for the non-paraplegic. So therefore...I would cure...the paraplegics.
Equality of life	Consideration of equal value of lives as a basis of moral judgement or to be fair.	If they're both living, it doesn't mean one's life is more valuable over the other.
Prejudice	Specific mention of wanting to avoid prejudice or discrimination.	Now I feel like I'm going to be prejudiced by picking this individual over that one.
		
**Self-Perspective**	Comments about self (or close-others) who have experienced the condition or projecting self into having the condition.	TTO – "...I would rather have pain on the leg than not be able to breathe properly."
		PTO – "I think I would have to try to choose the ones with the shortness of breath. ... my son had asthma pretty bad, ... I could live with the leg pain but I don't think I could live with the shortness of breath."

### Save-save group

The TTO elicitation reduced the number of years one friend would have to live in perfect health to be equally good as another friend who would live 50 years with paraplegia. We were interested in comparing responses to a PTO elicitation that asked how many people with paraplegia would need to be cured of a life-threatening infection to make them indifferent between curing that group versus curing 100 healthy people who had the life-threatening infection. Table 3 shows the median indifference point predicted from TTO responses and the median actual indifference point obtained through the PTO elicitation. The indifference point predicted from TTO responses was significantly higher than that obtained directly through the PTO elicitation. In the PTO elicitation, the median participant thought that curing the life-threatening infection in 100 people with paraplegia was equally good as curing the life-threatening infection in 100 healthy people. However, based on participants' responses to the TTO elicitation, we predicted that it would take saving 130 lives of people with paraplegia to be just as good as saving 100 healthy lives. In fact 91% of participants placed equal value on saving the lives of both groups when asked directly in the PTO elicitation but only 25% of TTO responses implied this value.

**Table 3:** Comparison of Predicted TTO and Actual PTO Indifference Points

Median (Interquartile Range) Indifference Points:			
Group	*Predicted from *TTO responses	Actual Response to PTO elicitation	*p^6^*
Save – Save^1 ^(n = 22)	130 (100–339)	100 (100–100)	0.001
Cure – Save^2 ^(n = 22)	176 (100–583)	135 (100–3250)	0.320
Cure – Cure^3 ^(n = 20)	323 (131-*infin^4^*)	650 (250-*infin^5^*)	0.736

1. The number of people with paraplegia whose lives would need to be saved to be equally good as saving the lives of 100 healthy people.

2. The number of people who would need to be cured of spinal cord injury to prevent paraplegia to be equally good as saving the lives of 100 healthy people.

3. The number of people who would need to be cured of moderate leg pain to be equally good as curing 100 people of severe shortness of breath.

4. Participants in the 75^th ^percentile believed that living 10 years with severe shortness of breath was equivalent to living less than a day in perfect health.

5. Participants in the 75^th ^percentile believed that all the people in the world would need to be cured of severe shortness of breath to be equally good as curing 100 people of moderate leg pain.

6. Based on the paired comparisons using the Wilcoxon signed rank test.

Why were the indifference points predicted from TTO responses larger than those obtained directly through the PTO elicitation? During the TTO elicitation, all participants voiced considerations that were consistent with QALY-maximization principles and only one participant mentioned a non-maximizing principle. In contrast, during the PTO elicitation, less than half (45%) mentioned QALY-maximization considerations, while two-thirds (68%) mentioned non-maximizing principles. The dominant consideration that emerged during the PTO elicitation was the belief that the lives of the people being traded-off were equal, regardless of pre-existing paraplegia. One man said, "I don't see leaning towards somebody because they can walk, valuing their life more than someone that would be in a wheelchair...Based on this description there isn't anything that says that I should lean towards one group or the other." Another man seemed to intertwine consideration of equality on the basis of saving a life and on the premise that the people with paraplegia were worse-off and should get higher weight, "They're already living life rough, you know what I mean, and then...not be able to save them?" Two participants wanted to avoid any taint of prejudice that may arise by choosing to cure the non-disabled group. One woman said, "I feel like I'm going to be prejudiced by picking this individual over that one." Another woman expressed strong emotion when asked to choose between curing the two groups, "Oh, this is horrible. This makes me feel like a bad person or something. I think I would choose the non-paraplegia... but...it doesn't make me feel good about myself...because these are people too and as many people may love them, as love these people. They may even have as many people dependent on them as these people." This participant later said, "...it's not different enough from saying 'Oh, well you're black or you're a woman or ... that kind of impermissible difference.' I guess [the two groups are] the same." Yet, this same participant was willing to trade-off years of life in exchange for perfect health when she responded to the TTO elicitation.

During the TTO elicitation, nearly half of participants (45%) took a personal perspective, imagining what it would be like to live in the condition themselves. Only two participants did so in the PTO elicitation. One man said, as he was responding to the TTO elicitation, "To have something like that happen to me, me personally ... if you only lived a day more. I wouldn't want to live like that" as he thought about living with paraplegia. Yet, many of the participants who personalized the scenario while responding to the TTO elicitation took a decidedly societal perspective in the PTO elicitation. The same man, as he responded to the PTO elicitation later said, "...They're already suffering...it's not fair to knock them off, you know what I mean? It wouldn't be right, it wouldn't be moral. If these people want to live like that, then you know what I mean? More power to 'em [sic]." One woman, after saying she'd prefer to live in perfect health even less than a day rather than live with paraplegia for 50 years in response to the TTO elicitation said, "When I think about having all your faculties up until the end, no matter what that point it is, I just prefer [living less than a day in perfect health]." However, later in the interview during the PTO elicitation, she said, "it's...like choosing to say who's life is more important – somebody who is fully functional or a person who is not. So, to me it's equal."

### Cure-save group

We presented the same TTO scenario to this group of participants as presented to participants in the Save-Save group. But the PTO scenario was different. The baseline of comparison in the PTO elicitation was curing 100 healthy people of a life-threatening infection and we asked participants how many people would need to be treated for a spinal cord injury to prevent the onset of paraplegia to make them indifferent about which group to treat. We found no significant differences between actual PTO indifference points and those predicted from TTO responses. During the PTO elicitation, the median participant believed that treating 135 people with a spinal cord injury to prevent paraplegia was equally good as saving the lives of 100 healthy people. The median indifference point predicted from TTO responses implied 176 people with spinal cord injury would need to be treated to be equivalent. Based on actual PTO indifference points, 45% of participants believed that curing spinal cord injury was at least as valuable as saving the life of a healthy person. This stance was also implied by 41% of the indifference points predicted from TTO responses.

When responding to the TTO elicitation, all participants voiced considerations related to QALY-maximization principles and most (68%) took a personalized perspective, as seen in Table 5. Likewise, when responding to the PTO elicitation, most participants (82%) voiced considerations consistent with QALY-maximization principles. One man weighed length of life versus quality of life as he responded to the PTO elicitation saying, "With the other ones ... they're not in as good condition because ... they're confined to a wheel chair, they're limited in what their activities are...I think I would go with [curing] the infection, just because, the fact is, that could result in death. These people [with paraplegia]: it would [be] nice to have them cured, so they can get around leading a normal life but without the treatment, they still are here ...They can still lead some sort of a life." Many participants focused on living with paraplegia compared to dying from an infection as one man who said, "...You'd like to live as long as you can...being paraplegic at least you're still on the planet where you can laugh and you can enjoy life and things like that" and a woman said, "So, at least they're alive, where if we hadn't helped these people, in 48 hours with this infection they would die."

**Table 5:** Percentage of Participants Coded for Each Theme

	Percentage of Participants:								
Group:	Save-Save (n = 22)			Cure-Save (n = 22)			Cure-Cure (n = 20)		
Topic	TTO	PTO	p*	TTO	PTO	p*	TTO	PTO	p*
*Topics consistent with QALY-maximization principles*									
Quality of life	91	36		95	55		100	95	
Length of life	55	5		55	55		50	5	
Non-health benefits	18	14		36	9		15	5	
	
Overall:	100	45	<0.001	100	82	0.13	100	95	1.00
									
*Topics not related to QALY-maximization principles*									
Concern for the worse-off	0	5		0	0		0	0	
Equality of life	5	59		5	0		0	10	
Prejudice	0	9		0	5		0	5	
	
Overall:	5	68	<0.001	5	5	1.00	0	15	0.25
									
Personalized the Scenario	45	9	0.004	68	27	0.01	45	25	0.10
% who said the TTO and PTO questions were different questions									
	64			59			35		

• Using McNemar's paired comparison test.

Many participants in this group implied that living with paraplegia was at least as bad as death by saying that living less than one day with perfect health was equally good as living 50 years with paraplegia as they responded to the TTO elicitation. Some participants continued to reflect this belief as they responded to the PTO elicitation by saying that treating people with a spinal cord injury to prevent paraplegia would be equally good as saving the lives of healthy people. One woman said, "...Both result in something totally life changing...because it results in death, but this is just really bad too...They just seem...equally as important..." This woman went on to say, "...What if I were an emergency room doctor or something, and somebody came in with this infection [and] somebody came in with this [spinal cord injury], which one would I take first and help...Both result in something totally life changing... [One] results in death, but this is just really bad too, so I don't know. They just seem...equally as important. Even though one results in death."

### Cure-cure group

The baseline of comparison for participants in the Cure-Cure group was curing 100 people of severe shortness of breath and we asked participants how many people would need to be cured of moderate leg pain to be equally good when responding to the PTO elicitation. The median participant believed that 650 people would need to be cured of moderate leg pain to be indifferent between treating the two groups when responding to the PTO elicitation. The median indifference point predicted from TTO responses implied that 323 people would need to be cured of moderate leg pain to be equivalent. Despite the seemingly large difference between the two values, it was not statistically significant. The non-significance is likely due to the exceedingly wide range of responses to both the TTO and PTO elicitations. Participants in the 75^th ^percentile, when responding to the TTO elicitation, would rather live less than a day in perfect health than live 50 years with severe shortness of breath. Similarly, participants in the 75^th ^percentile, when responding to the PTO elicitation, believed that every possible person should be cured of severe shortness of breath before anyone was ever cured of moderate leg pain. On the other end of the spectrum, two participants refused to trade any time in the TTO elicitation and two participants refused to choose a group to treat in the PTO elicitation; both sets of cases implied that curing either condition had equal value.

Our qualitative analysis revealed similar types of considerations across the two types of elicitations. All participants voiced considerations consistent with QALY-maximization when they responded to the TTO elicitation. One woman said, "I think that the shortness of breath would be...more suffering even though she's going to live longer and die peacefully." Another woman said, "I'd rather have pain in the leg than not be able to breathe properly." Naturally, because the TTO elicitation asked participants to trade-off years of life versus quality of life, directly, many more participants mentioned the importance of length of life as they responded to the TTO compared to the PTO elicitation saying, for example, "I think that a 30-year-old would value having more years of life, even if it involved having pain – daily pain."

All but one participant also voiced QALY-maximization considerations as they responded to the PTO elicitation too. One man said he would "still choose the shortness of breath because those people cannot function and the people with the leg pain can function; basically enjoy life with a little pain." Qualitatively, several participants said they wanted to be sure *everyone *was cured in the worse off group before anyone in the group with the less severe condition was cured. One man said, "The shortness of breath is...more severe...Moderate leg pain,... just about everybody has aches and pains. But that shortness of breath,...that's scary. So, I'd rather see everyone cured of the...shortness of breath." Another said, "I think that it would be more important to me to cure shortness of breath than to cure some moderate leg pain they may be having. I don't know, maybe I'm wrong to think that even six billion people, every person in the world had leg pain, I still think that it'd be more important to help the people with shortness of breath." A woman said, "To me it seems comparable to eliminating the common cold versus like getting rid of AIDS what would I choose? I would choose getting rid of AIDS...People die from that, they suffer terribly whereas this leg pain, it's a drag but it doesn't affect your whole life. Again, I would still cure one hundred and let the six billion deal with it."

Three participants (15%) voiced non-maximization principles while responding to the PTO elicitation, while none did so when responding to the TTO elicitation. Two participants specifically mentioned the importance of equality, but from different perspectives. One woman believed that everyone should have equal access to treatment, regardless of severity, "...Everybody is worthy of being alleviated from that pain." But another participant felt that everyone should have equal opportunity for a decent quality of life, "I feel like no matter what number I come up with for moderate leg pain, I'm gonna [sic] be left feeling guilty about ... all these people I know still have shortness of breath and can't make it from the bedroom to the bathroom". Another participant was concerned about inappropriately discriminating against treating a patient on the basis of their condition, "...They're both the same age, so you have to look at them equally...It's...like discriminatory if you put more weight towards the person that's going to die sooner,...if her death has nothing to do with the condition."

Nearly half of participants took a personalized perspective when responding to the TTO elicitation while only 25% did so as they responded to the PTO elicitation. When responding to the TTO elicitation, one man said, "I had leg pain ... I know some people that have shortness of breath and they can do a lot less than I can. They...might not have problems with their legs or anything but they can't...do anything. Walk a few steps, or else some of them have to carry their tanks around, their air tanks. So really, they're more hindered than...the person with the leg pain." He brought out considerations from a societal perspective as he responded to the PTO elicitation saying, "Well, it comes to a point, where would society be better off ... where you can cure more of the leg pain people, and then it would be more beneficial to society. I mean it sounds a little cruel but, part of those people could help take care of the people with severe shortness of breath."

### Perceived differences between TTO and PTO responses

When we asked participants to reflect on the indifference point we predicted from their TTO response compared the actual indifference point they gave in response to the PTO elicitation, we heard a wide spectrum of responses across the groups. In the Cure-Cure group, only 35% of participants felt the two elicitations were asking different questions, as shown in Table 5. However, most participants in the Save-Save and Cure-Save groups thought the elicitations were asking different questions (59% and 64%, respectively). One man said, "I think you still are worse off with paraplegia, right? You are definitely worse off, 'cause like I said, there's [sic] things you can't enjoy... If you're ... a politician or ... a public health person, I think you should value life either way ... even though you're worse-off with paraplegia." A woman said, "They're not the same question...You're asking me in the first one what is my perception of the illness...In that first question I took it as how would I feel if I had that illness or how would it affect me. Whereas this situation is asking me is how I value the two subsets as people. And as people, I value them equally. So one is a quality of life issue and the other one is who is more worthy of saving. They're not related at all." Some simply echoed one man who said, "I know the numbers don't match, but even looking at them now, I still would say the same thing." Some expanded a bit more as one woman did, saying that her TTO response was based on "Me personally...everybody doesn't think like I do," but as she talked about the PTO elicitation she said, "...One of the reasons is that both, without the treatment they'll both die. That's why I said they were 50/50. I couldn't decide to save one or the other... I had to pick both."

## Discussion

Based on our findings, non-maximizing principles explain part of the empirical differences seen in previous studies when values obtained from TTO responses were compared to those obtained from PTO elicitations. However, it is clear that context drives the extent to which these other principles come into play. Participants were clear in their belief that, within the context of choosing between life-saving treatments, having pre-existing paraplegia should not be a consideration. Though many participants took a decidedly personalized perspective when responding to the TTO elicitation, most did not carry this into the PTO elicitation and instead took a decidedly societal perspective. Even participants who preferred living less than a day in perfect health rather than live with paraplegia when responding to the TTO elicitation said that it was equally important to save the lives of both groups in the PTO elicitation. People were consistent in taking an egalitarian approach in the sense that everyone has a right to choose to live, regardless of their health condition. Our findings align with Williams's *fair innings *argument based on the belief that everyone deserves to live some normal length of life [39] and confirm Nord's [14] theory that people may feel free to make decisions about their own life but be reluctant to make life decisions for others. These findings are also empirically consistent with other studies where the median participant placed equal (or nearly equal) value on saving the lives of people with paraplegia or perfect health in PTO elicitations [22-24] and the importance of giving patients equal access to care [40].

Indifference points predicted from TTO responses were not significantly different from those obtained directly through the PTO elicitation when participants traded off curing spinal cord injury to prevent paraplegia versus saving a healthy life. Participants voiced considerations related to QALY-maximization principles while responding to both the TTO and PTO elicitation and they rarely mentioned other principles during either elicitation. Most previous studies comparing the PTO method to other traditional preference measures framed choices in terms of curing a less severe chronic condition than ones described in our study, versus saving the lives of previously healthy patients. In nearly all cases, contrary to our results, indifference points were higher when obtained through a PTO elicitation compared to those computed from a traditional utility elicitation method; this was true for the rating scale [18], visual analog scale [17], TTO [14,19,25], and standard gamble [14,17]. One reason for the lack of differentiation in our study may be because of the way we framed the PTO scenario. Rather than curing a pre-existing condition, we presented an opportunity to *prevent new onset *of paraplegia. Our findings do confirm a study done by the European Disability Weights group in which a substantial number (43%) of participants placed equal or higher value on preventing onset of quadriplegia compared to saving the lives of healthy people [41]. These findings may be related to the fact that the condition evaluated in the two studies (paraplegia or quadriplegia) involves severely impaired mobility. In two other studies, people also placed significantly less value on saving the lives of patients who would suffer new onset paraplegia compared to saving the lives of people with pre-existing paraplegia [22,23]. In a follow-up study, when participants were encouraged to consider their own ability to adapt to difficult situations, the relative valuation for a life-saving treatment with new onset paraplegia increased significantly [24]. This study provides qualitative confirmation that many people are focused on the initial trauma of having paraplegia in the context of this kind of elicitation. If people perceive the condition to be cured as highly traumatic, they will focus on the "awfulness" of the initial trauma such that, in the words of one of our participants, "both result in something totally life changing" when compared to the alternative of saving lives. There may be a theoretical threshold where as the condition to be cured becomes less severe, people focus on the imperative of saving lives instead. In this context, people's choices may become lexicographical when lives were at stake: lives typically trump cures [14,17-19].

Nearly all participants voiced only considerations that were consistent with QALY-maximization principles when asked to trade off years (TTO) to live in a less severe condition versus living longer with a more severe condition or when choosing which of two groups to cure (PTO) of a non-life-threatening condition. Indifference points predicted from TTO responses were comparable to indifference points obtained directly through the PTO elicitation. Some people think that more severely ill patients should be given higher priority even if they gain less benefit than treating less severely ill patients [4,18,19,22]. One study found that PTO valuations for curing patients who were worse-off were higher than those obtained from the rating scale [18]. Only one small-scale study compared the PTO to TTO preferences within the context of curing two non-life-threatening conditions. Dolan and Green found, qualitatively, that participants most often based their responses on what the difference in treatment benefit was likely to be [42]. Many of our participants expressed the belief that everyone should be cured of severe shortness of breath before anyone moderate leg pain was cured. As one participant stated, "if you've got a pain in the leg you can take an aspirin. If you can't breathe you can't breathe." Severe shortness of breath, the way we described it, was exceedingly more severe than the moderate leg pain we described. Taurek asserted that "the discomfort of each of a large number of individuals experiencing a minor headache does not add up to anyone's experiencing a migraine" [[43], page 308]. In the same vein, curing ever more people with moderate leg pain doesn't take away the suffering of a single person with severe shortness of breath. Several participants expressed this sentiment and this may help explain our findings. There may again be a threshold (working in the opposite direction) beyond which, if the difference in severity is pronounced enough, the PTO will result in higher indifference points than would be predicted by the TTO measure.

Nord [16] assumes that the PTO provides a social context within which to obtain preference weights and Olsen [15] maintains that the PTO implies social weights while the TTO implies private weights. Another study found qualitative evidence that some participants took a societal perspective in a PTO elicitation [42]. Our results confirm these assertions. Regardless of context, participants were more likely to take a personal perspective when responding to the TTO compared to the PTO elicitation. This is in spite of the fact that we framed the TTO elicitation from a non-personal perspective; we asked participants to evaluate two friends rather than imagine what it might be like to live with that condition personally. On the whole, participants in our study were still more likely to personalize the TTO compared to the PTO elicitation.

This study has several weaknesses. The sample size was small, with trends toward being unbalanced between the groups with respect to age and minority status. The actual indifference points are not generalizable because of the small convenience sample. In addition, our sample was limited to a Midwestern location in the U.S. The European Disability Weights study found that preferences elicited by a PTO scenario, similar to the one we presented to participants in the Save-Save group, varied widely across five European countries [41]. Additionally, we used a long time horizon (50 years) in the TTO elicitation to encourage more people to trade time for improved quality of life. However, indifference points may have been exceptionally high because people were more averse to living such a long time in the health condition being evaluated. This phenomenon may have contributed the lack of differentiation between TTO and PTO indifference points in two of three of the treatment contexts in our study though others have found differences in similar contexts. Despite differences in recruiting location, demographics, and structure of the interview, participants were consistent in their tendency toward taking a more personalized perspective when responding to the TTO compared to the PTO elicitation, regardless of context.

## Conclusion

When trading off groups of patients within the context of PTO elicitations, respondents are more likely to take a societal perspective than when trading time within the context of TTO elicitations – even when the TTO elicitation is framed in a non-personal way. Furthermore, when lives are at stake within the context of a PTO elicitation, people are more likely to consider principles other than simply maximizing QALYs, including the importance of equal access to a life-saving treatment, avoiding prejudice or discrimination, and in rare cases giving treatment priority based purely on the position of being worse-off. However, the extent to which these non-maximizing principles are expressed depends on context.

## Competing interests

Financial Disclosure: Financial support for this study was provided by the VA Ann Arbor Healthcare System, Ann Arbor, MI and NIH Grant #R01 HD40789. The funding agreement ensured the authors' independence in designing the study, interpreting the data, writing and publishing the report. The following authors are employed by the sponsor: Laura J. Damschroder and Peter A. Ubel. All funding agreements ensured the authors' independence in designing the study, interpreting the data, writing and publishing the report.

## Authors' contributions

LJD participated in design, implementation, led the qualitative and quantitative analyses, and drafted the manuscript. TRR participated in qualitative analyses of the data. CCG carried out the study and participated in the qualitative analyses. MEM participated in design and carrying out the study. PAU participated in design of the study, participated in qualitative analyses, and consulted on the analyses. All authors read and approved the final manuscript.

## Supplementary Material

Additional File 1 **Appendix 1 **PDF file with detailed elicitation text that was presented to participants for the TTO and PTO elicitations. Click here for file
