# Supplementary material for: Trading people versus trading time: What is the difference?
Source: Popul Health Metr. 2005 Nov 10;3:10. doi: 10.1186/1478-7954-3-10 (PMC1310516; doi:10.1186/1478-7954-3-10)
Supplement: Additional File 1 — Appendix 1 PDF file with detailed elicitation text that was presented to participants for the TTO and PTO elicitations. [file 1478-7954-3-10-S1.doc]

**APPENDIX**

TTO Scenario Used in Save-Save and Cure-Save Groups

Imagine that you have one friend, Mr./Mrs. Adams, who is 30 years old and has **paraplegia**. This means that he/she is paralyzed from the waist down. You can imagine that his/her bowel, bladder, and sexual function is normal, but he/she cannot move his/her legs at all, and has to use a wheelchair to get around. Mr./Mrs. Adams will live 50 more years with paraplegia, and then die in his/her sleep.

Now imagine that you have another friend, Mr./Mrs. Brown, who is 30 years old and in **perfect health**. This means he/she has no health problems at all. Mr./Mrs. Brown will live 50 more years in perfect health, and then die in his/her sleep.

Which friend do you think is better off or would you consider them to be equally well off? Remember to think out loud as you answer.

If the subject chooses Mr/Mrs. Brown, we ask, “Ok, imagine again that Mr./Mrs. Adams will live for 50 more years with paraplegia. Now imagine that Mr./Mrs. Brown will live for a few more days in perfect health. Which friend do you think is better off or would you consider them to be equally well off?”

TTO Scenario Used in Cure-Cure Group

Imagine that you have one friend, Mr./Mrs. Adams, who is 30 years old and has severe **shortness of breath**. S/he becomes severely short of breath even when walking only short distances, such as from the bedroom to the bathroom. Mr./Mrs. Adams will live 50 more years with this condition, and then die in his/her sleep.

Now imagine that you have another friend, Mr./Mrs. Brown who is 30 years old and has moderate **leg pain**, a condition caused by a narrowing of the arteries in the leg. S/he can walk a couple of blocks with no problem, but s/he feels pain in her/his leg whenever s/he walks uphill, or walks up several flights of stairs. Mr./Mrs. Brown will live 50 more years with moderate leg pain, and then die in his/her sleep.

Which friend do you think is better off or would you consider them to be equally well off? Remember to think out loud as you answer.

If the subject chooses Mr/Mrs. Brown, we ask: “Ok, imagine again that Mr./Mrs. Adams will live for 50 more years with shortness of breath. Now imagine that Mr./Mrs. Brown will live for a few more days with leg pain. Which friend do you think is better off or would you consider them to be equally well off?”

PTO Scenario Used in Save-Save Group (and as distractor in Cure-Cure Group)

Imagine that you are on a panel of experts trying to decide between two different medical treatments.

One treatment will cure people who have a **life-threatening infection**. If these people are not treated within 48 hours, they will **die**. With treatment, these people will be completely cured of their infection and they **will return to their former health**.

The other treatment will also cure people who have a **life-threatening infection**. This type of infection, however, only occurs **in people with paraplegia**, meaning that they are paralyzed from the waist down. If these people are not treated within 48 hours, they will **die.** With treatment, they will be completely cured of their infection and they will return to their former health, which means **they will** **remain paralyzed from the waist down**.

Imagine that you had to choose to cure 100 non-paraplegics of a life-threatening infection or to cure 100 paraplegics of a life-threatening infection. These groups are identical in every way except for their health. They are the same age, race, and gender, and have the same income. Which would you choose or are the two choices equally good?

Remember that our goal is to find the number of people who would need to be cured of each health condition so that the two choices seem equally good to you. Imagine that one choice is to cure 100 non-paraplegics of a life-threatening infection. How many paraplegics with a life threatening infection would have to be cured to make the two choices seem equally good to you?

PTO Scenario Used in Cure-Cure Group (and as second distractor task in Save-Save group)

Imagine that you are on a panel of experts trying to decide between two different medical treatments.

One treatment will cure people who have a **life-threatening infection**. If these people are not treated within 48 hours, they will **die**. With treatment, they will be completely cured, and they will return to their former health.

The other treatment will cure people who have suffered a **spinal cord injury**. If these people are not treated within 48 hours, they will be **left paralyzed from the waist down**. With treatment, they will be completely cured of their injury and they will return to their former health.

Imagine that you had to choose to cure 100 people of a life-threatening infection or to cure 100 people of a spinal cord injury that will result in paraplegia. These two groups are the same except for their health. For example, they are the same age, race, and gender and have the same income. Which would you choose or are the two choices equally good?

Remember that our goal is to find the number of people who would need to be cured of each health condition so that the two choices seem equally good to you. Again, imagine that one choice is to cure 100 people of a life-threatening infection. How many people would have to be cured of a spinal cord injury that will result in paraplegia to make the two choices seem equally good to you?

PTO Scenario Used for Cure-Cure Group (and as distractor task in Save-Save and Save-Cure Groups)

Imagine that you are on a panel of experts trying to decide between two different medical treatments.

One treatment will cure people who have severe **shortness of breath**. These people become severely short of breath even when walking only short distances, such as walking from the bedroom to the bathroom.

The other treatment will cure people who have moderate **leg pain**, a condition caused by a narrowing of the arteries in the leg. People with this condition can walk a few blocks with no problem, but they feel pain in their leg whenever they walk uphill or walk up several flights of stairs.

Imagine that you had to choose either to cure 100 people of severe shortness of breath or to cure 100 people of moderate leg pain. These two groups are the same except for their health. For example, they are the same age, race, and gender and have the same income. Which would you choose or are the choices equally good?

Remember that our goal is to find the number of people who would need to be cured of each health condition so that the two choices seem equally good to you. Again, imagine that one choice is to cure 100 people of shortness of breath. How many people would have to be cured of leg pain to make the two choices seem equally good to you?
